# Effects of carbon-fiber plate design on foot stress injury risk: a finite element analysis

**DOI:** 10.3389/fbioe.2025.1723984

**Published:** 2025-12-18

**Authors:** Linlin Guan, Yangyu Guo, Haichun Wang, Yusen Wu, Yunlong Jia, Xiaolan Zhu

**Affiliations:** 1 School of Sport Science, Beijing Sport University, Beijing, China; 2 Anta Sports Science Laboratory, Anta (China) Co., Ltd., Xiamen, China

**Keywords:** carbon-fiber plate, foot biomechanics, foot stress injury, metatarsal stress, soft tissue stress, structural design

## Abstract

**Introduction:**

Carbon-fiber plate (CFP) running shoes may alter ankle and foot biomechanical loading patterns, thereby potentially increasing the risk of foot stress injuries. However, there still a lack of systematic quantitative analysis of how multiple key design parameters (determining CFP performance) influence foot biomechanical responses. This study aimed to clarify how composite CFP influence the mechanical response of the foot and ankle, with the goal of reducing overuse injuries and providing biomechanical guidance for the structural design of CFP running shoes.

**Methods:**

A three-dimensional finite element (FE) model of the foot–CFP running shoes system was developed to analyze the effects of plate thickness (1.0, 1.25, and 1.5 mm), weaving type (Unidirectional carbon fiber (UD)/Woven carbon fiber (WO)), and ply angle (±30°, ±45°, and ±60°) on foot stress and midsole mechanical behavior.

**Results:**

The results showed that CFP thickness was positively related to midsole stiffness. Plantar stress first decreased and then increased with increasing plate thickness. A thicker plate reduced the peak metatarsal stress and led to a more even stress distribution across the forefoot. At the same thickness, UD plate with smaller ply angles lowered metatarsal loading, while WO plate with moderate angles also helped reduce stress concentration.

**Conclusion:**

During long-distance running, the thicker UD plate with a small ply angle exhibits greater potential for reducing loads on the metatarsals and plantar soft tissues, while the WO plate with moderate ply angle provides a more balanced load distribution. From the perspective of injury-risk reduction, the latter may be more favorable for recreational runners.

## 1 Introduction

Running is a popular form of exercise. As a prevalent cyclic movement, long-distance running places substantial mechanical loads on the human body. In particular, during the landing phase, the peak vertical ground reaction force (vGRF) can reach two to three times the body weight (Jiang et al., 2024). Elite runners often adopt a forefoot or midfoot strike pattern (Hayes and Caplan, 2012), which subjects the metatarsal region to frequent and high-intensity impacts. Over time, repetitive stress accumulation can lead to overuse injuries such as metatarsal stress fractures and metatarsalgia, which are particularly common among runners (Sobhani et al., 2014; Sun et al., 2024).

In recent years, CFP running shoes have been regarded as a key innovation for improving running performance and economy due to their increased longitudinal bending stiffness (Ortega et al., 2021). However, existing studies have not reached a consensus on the quantitative relationship between plate stiffness and running economy, reporting slightly decreased performance (Day and Hahn, 2020), no significant difference (Flores et al., 2019; Beck et al., 2020), or improvements of approximately 2.8%–4.2% (Hoogkamer et al., 2018; Barnes and Kilding, 2019; Hunter et al., 2019). The prevailing view suggests that enhanced longitudinal bending stiffness alters lower-limb biomechanics, thereby contributing to performance gains (Willwacher et al., 2014; Cigoja et al., 2019; Hoogkamer et al., 2019; McLeod et al., 2020). Meanwhile, the foot is the part of the lower-limb kinetic chain that is in direct contact with the CFP running shoe. Improper plate thickness or embedded position may lead to localized plantar pressure and elevated metatarsal stress, thereby increasing the risk of stress-related injuries (Song et al., 2023). The use of CFP running shoes has also been associated with navicular bone stress injuries (Tenforde et al., 2023). This further underscores the need to recognize that CFP footwear may alter biomechanical loading patterns, thereby increasing the risk of stress-related foot injuries.

At present, foot–shoe coupled finite element (FE) models have been used to evaluate the effects of sole structures on foot mechanical characteristics (Song et al., 2022; Yang et al., 2022; Wu et al., 2024). Previous studies have preliminarily explored the effects of the presence, thickness, and curvature of CFP on the foot (Song et al., 2023; Song et al., 2024). These studies indicate that CFP can optimize the load transfer pathways and make force distribution across the forefoot more uniform. Compared with shoes without a CFP, increasing stiffness or thickness was associated with decreased peak plantar forefoot pressure and reduced compressive strain under the forefoot, especially under low-load conditions. However, when the CFP is placed too high (or under certain conditions), some studies have reported increased peak metatarsal stress. Furthermore, curved CFP may reduce peak forefoot pressure more effectively than flat plates, although they do not significantly reduce stress in the more injury-prone second and third metatarsals. Meanwhile, the CFP mechanical performance is not determined by a single variable but rather by multiple structural parameters such as its thickness, fiber weaving type, and ply angle (Lee et al., 2014; Rahmani et al., 2014). However, systematic quantitative analyses remain lacking regarding how these key design parameters independently and interactively influence running shoe performance and foot biomechanical responses (Beck et al., 2020; McLeod et al., 2020; Song et al., 2024).

Based on this, the present study combines three-point bending simulations with FE analysis under peak ground reaction force during running to systematically investigate the effects of key structural parameters of the CFP (thickness, weaving type, and ply angle) on foot soft-tissue stress, metatarsal stress. The aim is to elucidate the underlying mechanisms by which plate parameters influence foot biomechanics, providing a theoretical basis for reducing the risk of stress-related foot injuries.

## 2 Methods

### 2.1 Biomechanical data acquisition and processing

The biomechanical data used in this study were obtained from a peer-reviewed public running dataset (Van Hooren and Meijer, 2024). In this dataset, muscle activations, fiber contraction velocities, and muscle forces were computed using OpenSim 3.3 and have been previously validated (Van Hooren et al., 2024). Among all participants, Subject No. 09 (male, 28 years old, height = 180 cm, body mass = 61.5 kg, foot length = 26.4 cm, rear-foot strike runner, comfortable running on a treadmill, forefoot sole thickness = 2 cm, heel thickness = 3.4 cm) showed the closest match to the anthropometric and footwear parameters used in the present model. Therefore, the kinematic and kinetic data from this subject running on a level treadmill at a speed of 3 m/s were selected as input for the simulations in this study (The protocol involved starting from zero speed, accelerating to 3 m/s, maintaining this speed for 1 min, and then decelerating back to zero).

**FIGURE 1:**
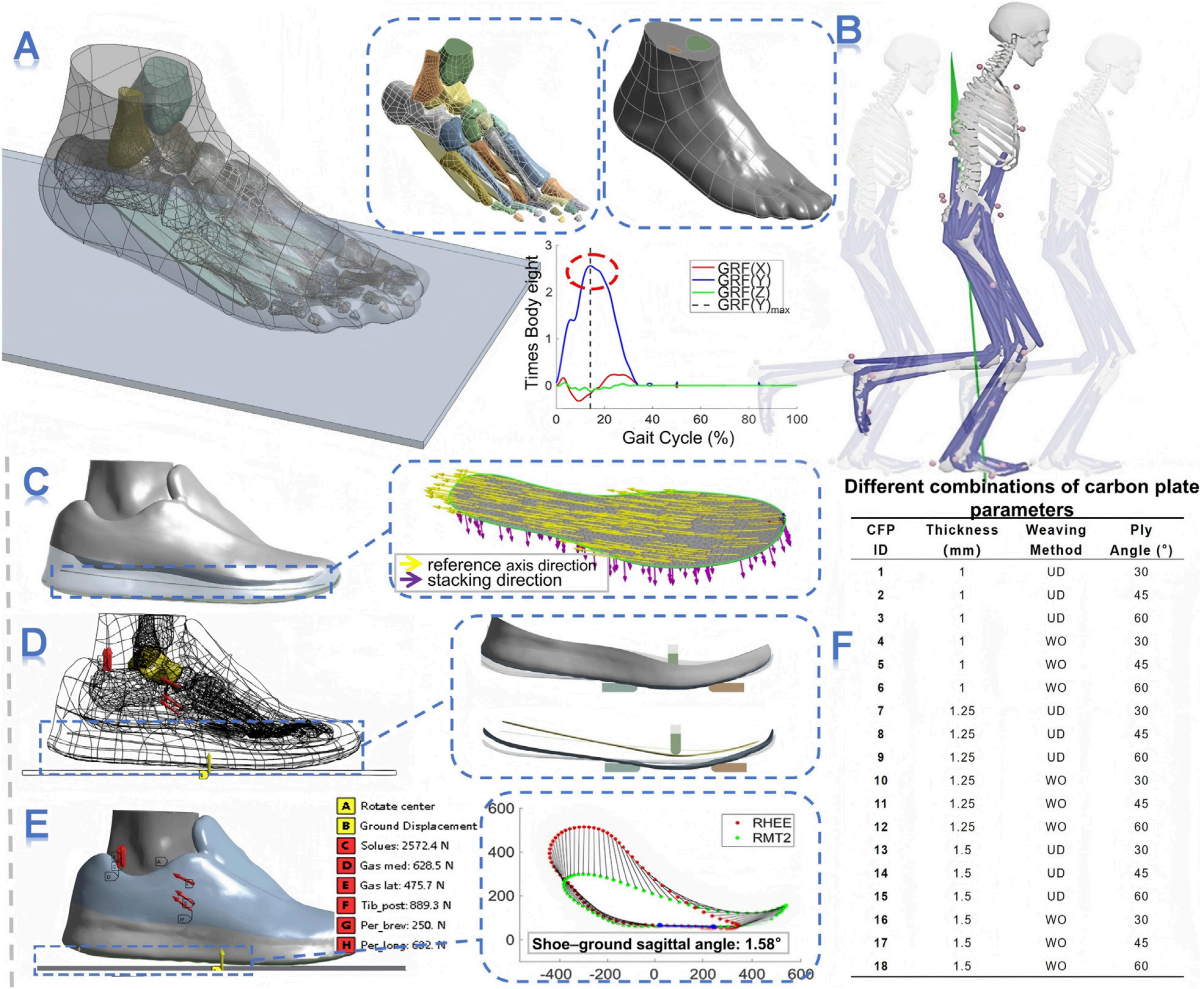
Research methodology **(A)** Construction of the foot–ankle finite element model; **(B)** Running simulation in OpenSim; **(C)** Modeling of the composite CFP (yellow vector: reference axis direction; purple vector: stacking direction); **(D)** Finite element simulation of the three-point bending test; **(E)** Finite element simulation at the peak moment of vertical ground reaction force during running; **(F)** Eighteen combinations of CFP parameters used in the simulations.

In this study, the Gait2354-Simbody musculoskeletal model in OpenSim 4.5 was used to reprocess the motion data (Figure 1B). The static calibration data and motion data files (.trc and. mot) of Subject 09 were imported into the software and sequentially processed through scaling (Scale), inverse kinematics (IK), inverse dynamics (ID), residual reduction algorithm (RRA), and computed muscle control (CMC). During the CMC analysis, the muscle forces of the ankle plantar flexors—including the soleus, medial gastrocnemius, lateral gastrocnemius, tibialis posterior, peroneus brevis, and peroneus longus—were extracted at the peak vGRF and applied as loading conditions to drive the FE model. Additionally, based on the trajectories of the heel marker (RHEE) and second metatarsophalangeal joint marker (RMT2), the shoe–ground sagittal plane angle at the instant of peak vGRF was calculated (Figure 1E) and used as a positional constraint in the model.

### 2.2 Construction of the foot–CFP running shoe FE model

The foot–shoe FE model used in this study (Figure 1A) was derived from a healthy male subject (25 years old, 168 cm tall, 67 kg body weight, and foot length = 25.5 cm, rear-foot strike runner, comfortable running on a treadmill). The footwear was a pair of conventional running shoes (forefoot thickness: 2 cm; heel thickness: 3 cm) provided by Anta Co., Ltd. (Xiamen, China). This model has been validated and applied in our previous work (Zhu et al., 2023). The original foot model featured a high level of anatomical detail, including one soft tissue, 29 bones, 28 cartilages, five plantar fasciae, and 108 ligaments. The running shoe model consisted of three components—the upper, midsole, and outsole. Based on this structure, a CFP surface model was constructed in ANSYS DesignModeler (Figure 1C) (Guo et al., 2025). In the ANSYS material database, two typical prepreg layups used in composite design were selected: UD and WO carbon fiber prepregs. The UD layup features fibers almost entirely aligned in a single direction, providing high load-bearing capacity along the fiber axis but low strength and stiffness perpendicular to it. Conversely, the WO layup is composed of two interlaced fiber sets, with fibers oriented in two directions within each layer, resulting in more balanced mechanical properties (Amsalu et al., 2024). Subsequently, in ANSYS Composite PrepPost (ACP) (Asiri, 2022; Reda et al., 2024), two carbon fabric plies (each 0.125 mm thick) were combined to form a single laminate layer with a total thickness of 0.25 mm. The fiber orientations of the two plies were set at ±30°, ±45°, and ±60° relative to the reference axis (Mohamed and Abdelbary, 2023). According to the typical thickness range of CFP used in commercial running shoes, four-, five-, and six-layer laminate structures were designed to obtain CFP with total thicknesses of 1.00 mm, 1.25 mm, and 1.50 mm (Song et al., 2024), respectively. The resulting composite CFP models were subsequently used for FE analysis (Figure 1F).

In terms of material properties, the specific material parameters of the foot–CFP running shoe–ground FE model and the fixtures used for the three-point bending test of the shoe sole are listed in Table 1 (X, Y, Z refer to the three orthogonal material directions (fiber direction, in-plane transverse direction, and thickness direction); XY, YZ, XZ refer to the corresponding material planes for Poisson’s ratios and shear moduli). The material properties of each foot-tissue (Zhu et al., 2023) and shoe component (Guo et al., 2025) were selected from the literature. For the contact definitions in our foot–CFP running-shoe–ground FE model, we defined three contact pairs—soft tissue–shoe upper, soft tissue–midsole, and outsole–ground—as frictional contacts, using a friction coefficient of 0.6, while all other contact pairs were set as bonded contacts (Zhu et al., 2023; Wu et al., 2024). In the three-point bending FE model, the contact between the loading head and the midsole, as well as that between the supporting frame and the outsole, were also defined as frictional contacts with a friction coefficient of 0.6 (Wu et al., 2024), and the remaining contacts were set as bonded. Both models adopted Solid187 structural solid elements, and meshing was performed using a tetrahedral patch conforming method. Local mesh refinement and sensitivity analysis were conducted in the contact regions between the soft tissue and the midsole. Following previous literature (Abdullah et al., 2025), we considered the mesh converged when the difference between outputs from two successive mesh densities was less than 5%. Based on a balance among mesh quality, computational efficiency, and numerical accuracy, we selected the current mesh sizes. The mesh sizes were set to 15 mm for the shoe upper and ground, 8 mm for the soft tissue (4 mm for the plantar soft tissue), midsole, and outsole, and 6 mm for the foot bones. The foot mesh comprises 75,825 elements and 131,579 nodes. The mesh and node counts for the shoe vary with CFP thickness: for the 1.0 mm CFP 10,773 elements/17,944 nodes for the carbon plate, and 30,820 elements/54,119 nodes for the shoe. For the 1.25 mm CFP: 13,167 elements/22,430 nodes for the carbon plate, and 33,214 elements/58,605 nodes for the shoe. For the 1.5 mm CFP: 15,561 elements/26,916 nodes for the carbon plate, and 35,608 elements/63,091 nodes for the shoe.

**TABLE 1:** Material properties of the FE model.

Material	Density (kg/m^3^)	Young’s modulus (MPa)	Poisson’s ratio	Shear modulus (MPa)
UD plate	1490	X:121000	XY:0.27	XY:4700
		Y:8600	YZ:0.4	YZ:3100
		Z:8600	XZ:0.27	XZ:4700
WO plate	1420	X:61340	XY:0.04	XY:3300
		Y:61340	YZ:0.3	YZ:2700
		Z:6900	XZ:0.3	XZ:2700
Soft tissue	937	1.15	0.49	—
Foot bone	1500	7300	0.3	—
Plantar fascia	937	350	0.45	—
Upper sole	2300	11.76	0.35	—
Midsole	2300	2.49	0.35	—
Outsole	230	8	0.47	—
Ground	5000	17000	0.1	—
Fixture (steel)	7850	200000	0.3	—

### 2.3 Three-point bending and running simulation

The three-point bending simulation used in this study was validated in our previous research (Figure 1D) (Guo et al., 2025). The loading head was positioned directly above the centerline of the supporting frame, corresponding to the metatarsophalangeal joint bending region of the shoe sole (approximately 73% of the total sole length) (Fraser et al., 2014). After the shoe sole was clamped and fixed within the fixture, the loading head was driven downward at a speed of 10 mm/s for the simulation test (Takahashi et al., 2016). The nonlinear transient three-point bending simulation of the sole was performed in the Transient Structural module of ANSYS Workbench (version 2019, ANSYS Inc., Canonsburg, PA, United States). Regarding boundary conditions and loading, the supporting frame at the bottom was constrained as fixed, while a vertical displacement of 10 mm was applied to the loading head (Table 2). The total simulation duration was set to 1 s. The 80%–90% segment of the loading head’s force-displacement curve was extracted and linearly fitted via MATLAB (R2024a, MathWorks, Natick, MA, United States). The slope of this equation was defined as the sole’s longitudinal bending stiffness (Cigoja et al., 2019). The fitting equation is as follows:

F=K·d+b

**TABLE 2:** Boundary conditions for three-point bending and running simulation.

Simulation type	Loading method		Constraint conditions	
	Parts	Setting	Parts	Setting
Three-point bending	Loading head	Downward displacement 10 mm	Support frame	All degrees of freedom constrained
Running simulation	Ground	Upward displacement 3.2 mm	Foot–shoe	Sagittal plane angle of 1.58°
	Peroneus longus	2572.4N	Talus	Only allows rotation around X-axis (ankle joint flexion–extension); other degrees of freedom constrained
	Medial gastrocnemius	628.5N		
	Lateral gastrocnemius	475.7N		
	Soleus	889.3N		
	Peroneus brevis	250N		
	Peroneus longus	632N		

In the equation, F represents the applied force (N), d denotes the displacement (mm), K is the slope, which corresponds to the bending stiffness (N/mm), and b is the intercept on the y-axis.

For the running simulation (Figure 1E), a nonlinear quasi-static analysis was conducted at the instant of the peak vGRF using the Static Structural module in ANSYS Workbench. A local coordinate system was established based on the centroid of the talus to define the ankle joint, with only rotation about the X-axis permitted to simulate ankle plantarflexion–dorsiflexion motion, while the remaining five degrees of freedom were constrained. According to the anatomical origins and tendon paths of the ankle plantarflexor muscles, the points of force application and directions of each muscle force were defined (Table 2) to construct the ankle plantarflexion moment. A vertical displacement load was then applied to the model such that the resultant reaction achieved mechanical equilibrium with the generated plantarflexion moment (Yang et al., 2022; Song et al., 2023; Song et al., 2024).

## 3 Results

### 3.1 Three-point bending simulation results

**FIGURE 2:**
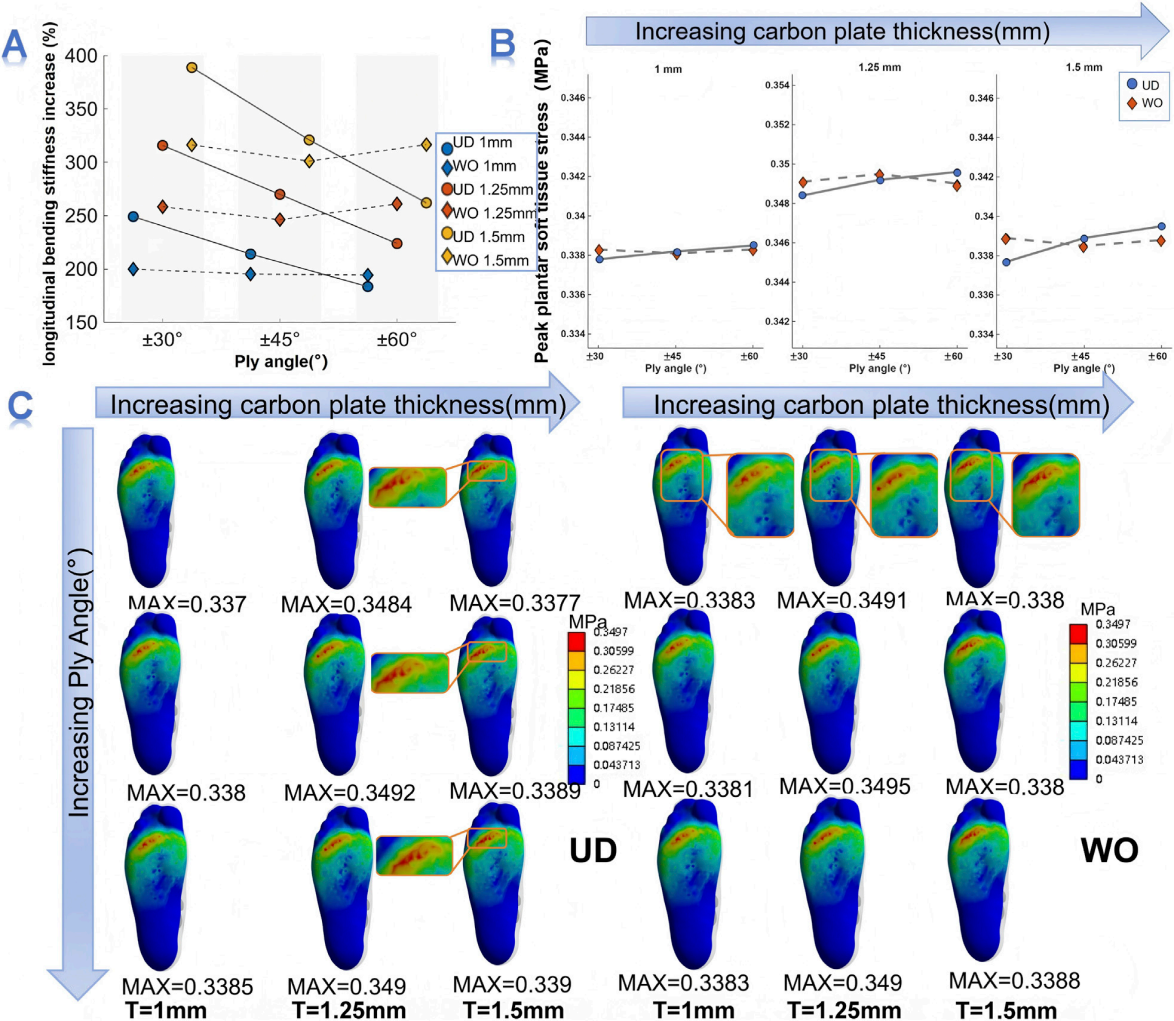
Effects of CFP parameters on shoe sole bending stiffness and plantar soft tissue stress. **(A)** Influence of CFP parameters on the longitudinal bending stiffness of the shoe sole. **(B)** Effects of CFP parameters on plantar soft tissue stress magnitude. **(C)** Effects of CFP parameters on the distribution of plantar soft tissue stress.

Under identical weaving type and ply angle, the sole stiffness increased with the CFP thickness (Figure 2A). Taking the UD plate with a ±45° ply angle as an example, when the plate thickness increased from 1.0 mm to 1.5 mm, the longitudinal bending stiffness of the sole increased by 214.08%, 269.85%, and 320.84% (the no-plate sole serves as the baseline condition), respectively. At the same thickness, the effect of ply angle on bending stiffness was influenced by the weaving type. The bending stiffness of the UD plate was more sensitive to changes in ply angle: as the ply angle increased from ±30° to ±60°, the stiffness showed a monotonic decrease. In contrast, the WO plate exhibited relatively minor variations with ply orientation. At a thickness of 1.25 mm, the stiffness enhancement of the UD plate decreased from 315.70% to 269.85% and then to 223.90%, while that of the WO plate fluctuated slightly between 246.26% and 260.99%. Notably, the WO plate consistently exhibited the lowest stiffness enhancement at a ±45° ply angle, with increments of 195.32%, 246.26%, and 301.03% for the three thickness levels. Furthermore, the influence of the weaving type on longitudinal bending stiffness also depended on the ply orientation. At ±30° and ±45°, the UD plate showed higher bending stiffness than the WO plate, whereas at ±60°, the WO plate surpassed the UD plate.

### 3.2 Running simulation results

The CFP thickness exhibited a pronounced effect on the mechanical characteristics of plantar soft tissue stress (Figure 2B). Specifically, in terms of magnitude, as the plate thickness increased from 1.0 mm to 1.25 mm, the peak plantar soft tissue stress increased for all weaving types and ply orientations, rising from 0.3378–0.3385 MPa to 0.3484–0.3496 MPa. When the thickness was further increased from 1.25 mm to 1.5 mm, the peak stress decreased to 0.3377–0.3395 MPa. Across all three thickness conditions, the peak plantar soft tissue stress of the UD plate increased with larger ply angles, indicating that the smallest stress occurred at ±30° under the same thickness. In contrast, the WO carbon plate was less sensitive to changes in ply orientation. The influence of weaving type on peak plantar stress also depended on ply orientation. At ±30°, the UD plate consistently produced lower peak stress than the WO plate, whereas at ±60°, the UD plate produced higher values. When the ply angle is ±45°, the peak plantar soft tissue stress of the UD plate was approximately the same as that of the WO plate. Regarding the spatial distribution of plantar stress, the high-stress region was mainly concentrated in the forefoot, particularly beneath the second to fourth metatarsophalangeal joints. This distribution pattern remained consistent across all carbon plate configurations. However, it is noteworthy that the concentrated stress beneath the metatarsophalangeal joints became more dispersed as the carbon plate thickness increased (Figure 2C).

**FIGURE 3:**
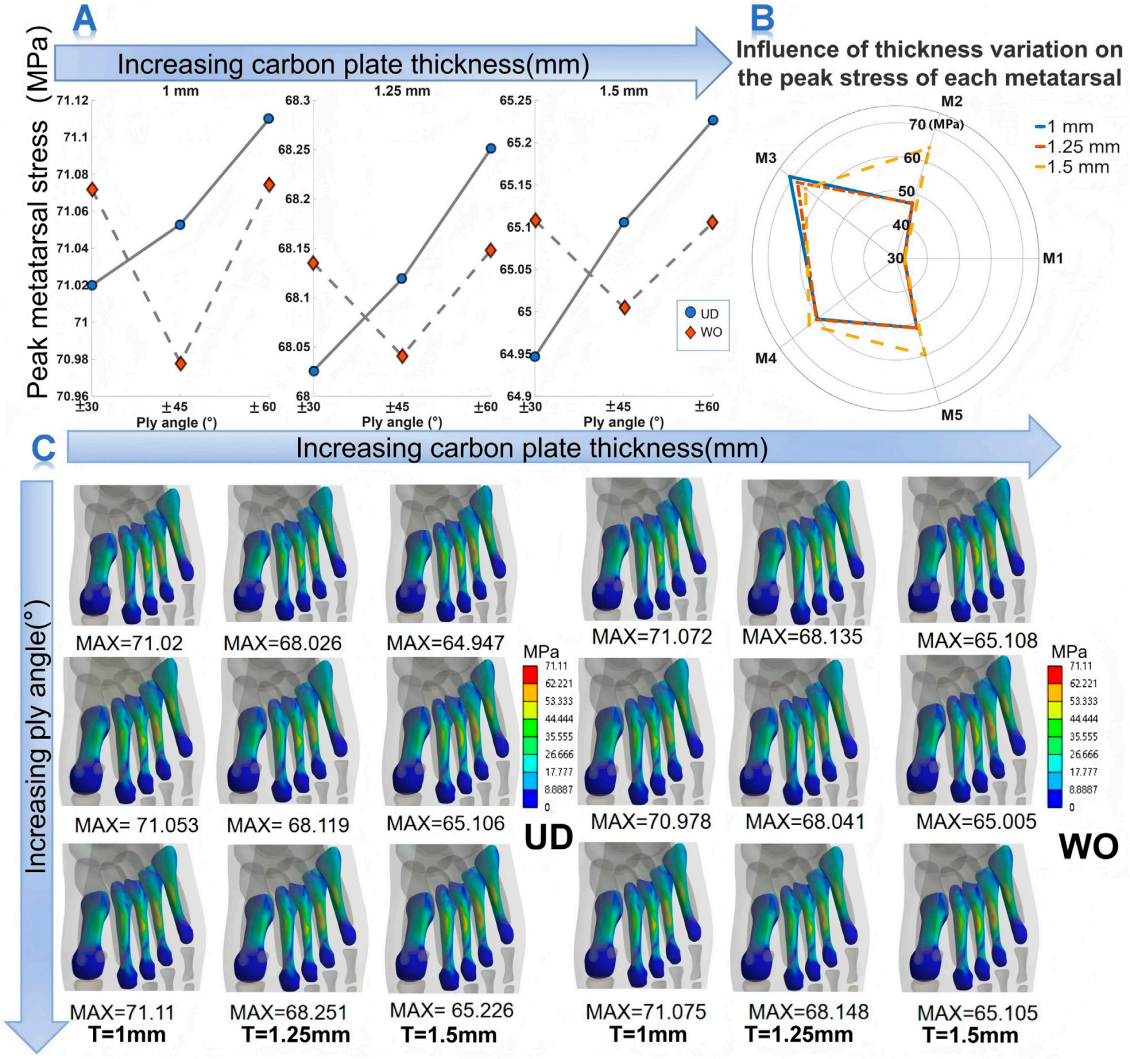
Effects of CFP structural design on metatarsal stress. **(A)** Effects of CFP weaving types and ply angle on peak metatarsal stress. **(B)** Effects of CFP thickness on peak metatarsal stress. **(C)** Effects of CFP parameters on the distribution of metatarsal stress.

Changes in CFP parameters affected the magnitude and distribution of metatarsal stress (Figure 3). First, for both UD and WO CFPs, the peak metatarsal stress showed a decreasing trend with increasing plate thickness across the three thickness conditions. Compared with the 1.00 mm UD plate, the peak metatarsal stress of the 1.25 mm UD plate decreased by 4.02%–4.22%, and that of the 1.50 mm UD plate decreased by 8.28%–8.56%. For the WO plates, the reduction range was more stable: the 1.25 mm WO plate showed a decrease of 4.12%–4.14%, and the 1.50 mm WO plate showed a decrease of 8.40%–8.42% (Table 3). Second, the influence of fiber angle on the peak metatarsal stress varied depending on the weaving type. For the UD plates, the peak metatarsal stress increased with the fiber orientation angle under all three thickness conditions. For the WO plate, the lowest peak metatarsal stress occurred at a fiber orientation of ±45°, while the values at ±30° and ±60° were similar and both higher than at ±45° (Figure 3A). The effect of the weaving type on the peak metatarsal stress also changed with the fiber orientation. At the same thickness, the peak metatarsal stress of the UD plate was lower than that of the WO plate at ±30°, but the opposite was true at the other two angles. In terms of stress distribution characteristics, across all three thicknesses, the stress peak consistently appeared in the third metatarsal shaft, followed by the fourth metatarsal, then the second and fifth metatarsals, while the first metatarsal experienced the lowest stress. However, as the plate thickness increased, the stress concentration in the third metatarsal gradually diffused toward the second, fourth, and fifth metatarsals (Figures 3B,C).

**TABLE 3:** Peak metatarsal stress (MPa) under different carbon plate parameters.

Weave type	Thickness	±30°	±45°	±60°
UD	1 mm	71.02	71.053	71.11
	1.25 mm	68.026 (↓ 4.22%)	68.119 (↓ 4.13%)	68.251 (↓ 4.02%)
	1.5 mm	64.947 (↓ 8.56%)	65.106 (↓ 8.37%)	65.226 (↓ 8.28%)
WO	1 mm	71.072	70.978	71.075
	1.25 mm	68.135 (↓ 4.13%)	68.041 (↓ 4.14%)	68.148 (↓ 4.12%)
	1.5 mm	65.108 (↓ 8.4%)	65.005 (↓ 8.42%)	65.105 (↓ 8.4%)

## 4 Discussion

This study employed a three-point bending simulation system to evaluate the regulatory mechanism of longitudinal bending stiffness in shoe soles under different CFP structural designs. Furthermore, by integrating experimental data from sports biomechanics and a musculoskeletal multibody dynamics model, FE simulations were conducted to quantitatively analyze the effects of various CFP parameters on plantar soft tissue and metatarsal stress. The findings not only elucidate the mechanical regulation of key CFP parameters on the foot–shoe system but also provide biomechanical evidence and engineering guidance for optimizing the structural design of CFP running shoes, aiming to reduce foot injury risks.

The bending stiffness of running shoe soles is a key indicator of shoe performance, reflecting the sole’s resistance to bending and closely relating to running economy, lower-limb biomechanics, and the risk of foot injuries (Ortega et al., 2021; Chen et al., 2022; Song et al., 2023). Three-point bending simulations revealed that different CFP design parameters noticeable influence the longitudinal bending stiffness of the sole, with notable interaction effects. CFP thickness was identified as the primary determinant of sole stiffness, with increased thickness directly enhancing bending resistance (Guo et al., 2025). More importantly, this study demonstrated a noticeable interaction between the weaving type and ply angle. In this study, UD refers to two-dimensional unidirectional weaving, with fibers primarily aligned in a single direction, while WO refers to two-dimensional woven weaving, with fibers interlaced in two directions (Ekşı and Genel, 2017). Due to the concentrated fiber orientation in UD plates, the projected stiffness contribution is maximized when the ply angle aligns with the principal loading axis. As the angle deviates from the principal axis, the bending stiffness noticeably decreases—a trend that becomes more pronounced with increasing angle, consistent with previous findings (Parmiggiani et al., 2021). This effect is amplified with increasing plate thickness, likely because additional layers magnify the influence of ply orientation on UD plates. In contrast, WO plates, with their orthogonal fiber structure, distribute loads more evenly and exhibit greater robustness to changes in ply angle (Belliveau et al., 2022). At larger angles (±60°), WO can even achieve higher bending stiffness than UD. These findings indicate that coordinated manipulation of the “thickness–weaving–angle” triad enables precise and targeted regulation of sole stiffness. This approach allows fine-tuning of bending stiffness without altering plate thickness (and thus weight), providing a novel strategy for lightweight design (Chang et al., 2024).

Another noteworthy finding is that, within the range of parameters selected in this study, the peak plantar soft tissue stress exhibited a nonlinear trend with increasing CFP thickness, initially rising and then decreasing. Tissue stress reflects the integrated stress state of biological structures and is particularly important for understanding the mechanisms of stress-related injuries (Chen et al., 2010). Previous studies have suggested that increasing the CFP thickness can reduce peak plantar soft tissue stress and alleviate localized stress concentrations (Song et al., 2024). In this study, the nonlinear changes in plantar soft tissue stress are likely influenced by the combined effects of CFP structural parameters, highlighting the complexity of CFP design. When CFP thickness increases from 1.0 to 1.25 mm (low to medium thickness), bending stiffness increases, and local pressure is transmitted to a concentrated forefoot area—slightly elevating soft-tissue stress. However, when thickness further increases from 1.25 to 1.5 mm, the overall stiffness becomes sufficiently high, enabling more uniform force transmission and deformation support in the sole. Thus, forefoot pressure distribution becomes more diffuse, and the soft-tissue stress decreases. At the same time, given individual differences among participants, future studies could include a more diverse range of participant types to address potential variability. Compared with the effect of thickness on plantar pressure, the weaving pattern and ply angle primarily serve a modulatory role. Under the same plate thickness, UD plates with fibers laid at ±30° exhibited higher stiffness, which helped distribute pressure and further reduce soft tissue stress (Song et al., 2024). In contrast, WO plates showed minimal variation in peak pressure across different ply angles, demonstrating better stiffness balance and more stable load distribution. During long-distance running, peak pressures in the forefoot region are high (Bowser et al., 2018; Matijevich et al., 2019), and chronic accumulation may lead to overuse injuries such as soft tissue fatigue and metatarsal stress fractures (Kakouris et al., 2021). Existing footwear intervention studies indicate that a more uniform pressure distribution is considered an effective strategy to reduce potential injury risk (Melia et al., 2021). Therefore, adjusting carbon plate parameters to modulate plantar stress may provide a feasible approach for lowering the risk of foot overuse injuries.

Metatarsal stress fractures are common among individuals engaged in repetitive weight-bearing activities, with the second or third metatarsals being particularly susceptible in long-distance runners (Sun et al., 2024). In this study, increasing the CFP thickness from 1 mm to 1.5 mm significantly reduced peak metatarsal stress and promoted a more uniform stress distribution across the metatarsals, thereby alleviating stress concentration and lowering the risk of injury (Song et al., 2024). Consistent with the trends observed in the three-point bending tests, the weaving pattern and ply angle of the CFP also played important modulatory roles. Under the same thickness, a UD plate with a small ply angle reduced peak metatarsal stress more effectively than plates with larger ply angles. For WO plates, a ply angle of ±45° also contributed to lowering peak metatarsal stress, likely due to the combined effects of CFP parameters, which enhanced stress balance. It is noteworthy that changes in plantar soft tissue stress did not correspond directly to changes in metatarsal stress. The foot, as a complex biomechanical system, experiences stress influenced by multiple factors, including bone structure, load transfer through soft tissues, material properties, and load direction (Sigward and Powers, 2006). Furthermore, redistributing forefoot bending moment may alter plantar soft-tissue stress and metatarsal stress in ways that are not directly correlated. Therefore, when evaluating the protective performance of running shoes, the mechanical responses of both bones and soft tissues should be considered integratively, rather than inferring internal bone loading solely from external pressure distribution. From the perspective of reducing metatarsal stress-related injuries, within the parameter range examined in this study, a CFP with 1.5 mm thickness, a ±30° ply angle, and unidirectional weaving can effectively lower peak metatarsal stress and promote a balanced stress distribution.

The limitations of this study are as follows. First, although the musculoskeletal and FE models used have been validated, from an application perspective, the demands on shoe performance may vary across different running tasks and populations; Therefore, the results may be influenced by individual differences. Second, considering the complexity of the foot structure, the FE model in this study was simplified to improve computational efficiency, but this may reduce model accuracy. The constraint on ankle degrees of freedom may have affected the mechanical response of the talus. In future work, we could consider allowing more ankle-joint freedom. And in future work we could perform dynamic simulations to better approximate running conditions. In the current model both bone and soft tissue are assumed to have homogeneous material properties. While this assumption simplifies the model and reduces computational demand, it may neglect the known heterogeneity and anisotropy of biological tissues. Third, the range of variables selected in this study was relatively narrow. Future studies could consider expanding the variable range. And we recognise that emerging methodological advances—such as statistical shape modeling (SSM), free-form deformation (FFD), and deep learning based surrogate modeling for FE analysis—provide promising approaches to address these limitations. For example, recent studies have demonstrated that SSM combined with FFD can efficiently generate personalized 3D foot–ankle models from surface scans, capturing anatomical variation across a population. Deep learning surrogates have also shown potential to dramatically accelerate FE analysis workflows while maintaining acceptable accuracy, enabling efficient exploration of parameter spaces and population variability (Song et al., 2025).

## 5 Conclusion

Within the parameter range of this study, the combination of CFP thickness, weaving type, and ply angle enables precise control of running shoe sole longitudinal bending stiffness. UD plates with small ply angles effectively enhance stiffness, while WO plates provide more stable performance across different angles. Through simulation of the peak GRF moment during running, we find that CFP parameters exhibit a combined effect on foot biomechanics, with plantar soft-tissue stress showing a nonlinear relationship with plate thickness. Increasing plate thickness reduces peak metatarsal stress and promotes a more uniform stress distribution. At the same thickness, small-angle unidirectional plates reduce metatarsal loading, whereas bidirectional plates at moderate angles also alleviate metatarsal stress. Moreover, the thicker the plate, the more effective a unidirectional plate is in reducing metatarsal load. Overall, within the parameter range of this study, a thicker UD plate with a small ply angle is optimal for reducing metatarsal and plantar soft-tissue loading in long-distance runners, while a WO plate with a moderate ply angle offers a compromise between stability and comfort, potentially making it more suitable for recreational runners.

## Data Availability

The original contributions presented in the study are included in the article/supplementary material, further inquiries can be directed to the corresponding author.
